# Decreased sleep quality in high myopia children

**DOI:** 10.1038/srep33902

**Published:** 2016-09-21

**Authors:** Masahiko Ayaki, Hidemasa Torii, Kazuo Tsubota, Kazuno Negishi

**Affiliations:** 1 Department of Ophthalmology, Keio University School of Medicine, Tokyo, Japan

## Abstract

The aim of the present study was to evaluate sleep quality in myopic children and adults. This cross sectional study surveyed 486 participants aged from 10 to 59 years with refractive errors using a questionnaire containing the Pittsburgh Sleep Quality Index (PSQI) and Hospital Anxiety and Depression Scale (HADS). Children (< 20 years) in the high myopia group exhibited the poorest PSQI scores (*P* < 0.01), while the adults showed no such correlations. Subscales of PSQI and HADS in children disclosed that the high myopia groups had the shortest sleep duration (*P* < 0.01), worst subjective sleep scores (*P* < 0.001), and latest bedtime (*P* < 0.05). Regression analyses in children significantly correlated myopic errors with PSQI (*P* < 0.05), sleep duration (*P* < 0.01), and bedtime (*P* < 0.01). Sleep efficacy (*P* < 0.05) and daytime dysfunction (*P* < 0.05) were significantly better in contact-lens users compared to the respective non-user groups across all participants. In conclusion, sleep quality in children was significantly correlated with myopic error, with the high myopia group worst affected.

Myopia is a serious eye-health problem worldwide that particularly affects Asian populations^1^,^2^,^3^. It is often complicated by retinal detachment, macular degeneration, glaucoma, and cataract, although the only systemic associations documented for myopia are higher risks of sleep disorders and depression^4^,^5^,^6^. Sleep problems in children are also an emerging issue in Asia, with lower school performance recently associated with frequency of poor sleep^7^. Indeed, nighttime exposure to ambient light^8^,^9^,^10^ and various lighting displays^11^,^12^,^13^ were proposed as environmental hazards for sleep. In terms of myopic progression, outdoor activity was recently established as an antimyopiagenic factor^14^,^15^,^16^,^17^,^18^ in addition to known ones including age, genetic predisposition, urbanization, and near work^19^. The effect of school lighting on myopic progression is also a potential new factor under investigation^20^.

Retinal damage and stretch are common pathological features in myopia, presumably causing damage to the intrinsically photosensitive retinal ganglion cells (ipRGCs), which were shown in animals to modulate ocular growth and myopia progression via photoreception of short wavelength light^21^,^22^. Thus, this newly discovered non-visual photoreceptor in the RGC layer^23^,^24^,^25^ could potentially affect the risk of sleep disorders in myopic subjects with retinal damage such as glaucoma^26^,^27^,^28^. Blindness and cataract have also been associated with disorders of sleep and circadian rhythms since these disorders also involve defects in light transmittance and photoreception^29^,^30^,^31^. However, despite myopia being a very common ocular status, the sleep status in such patients has not been extensively evaluated.

Myopia is a potential cause of sleep disorders (Fig. 1), with affected persons showing poor unaided vision accompanied by extensive retinal damage and neuronal dysfunction^32^,^33^,^34^, although the status of ipRGCs in human myopia remains to be determined. In addition, dependence on optical correction devices (spectacles and contact lens) can cause serious distress, especially for high myopia persons, because these devices become a lifeline to sight and thus quality of life (QOL). It is a well known fact that sleep quality is worsened in depressive subjects^35^; indeed, this could be the case with sleep quality in myopes with nonsurgical optical correction with declined QOL in general health and mental health^36^.

In children particularly, many factors can influence the timing, duration, and quality of sleep including age, circadian timing, societal constraints such as school pressures, and the sleep environment^37^,^38^,^39^,^40^. Children are also not free from social jet lag^41^ even under scheduled weekday programs, and can experience delayed intrinsic sleep timing concurrent with the demands of early school-start times that lead to circadian misalignment. Nevertheless, healthy children qualify as very suitable candidates for studying myopia and sleep because they have clear optic media and more active photoreception than adults^42^,^43^. In addition, myopia shows the most rapid progression in younger individuals. Thus, comparative studies of sleep among different age groups might reveal a correlation between refractive error and ocular or systemic health; however, to the best of our knowledge, no such study has been conducted for humans with myopia. Therefore, we initiated a cross sectional study to evaluate sleep in myopic children and adults, and the potential flow-on effects on mood disorders and general health.

## Methods

Six eye clinics across various practices and locations participated in this study. A suitably constituted Ethics Committee of the Department of Ophthalmology, Keio University School of Medicine approved this research project, which conforms to the provisions of the Declaration of Helsinki, 1995 (as revised in Edinburgh 2000). Informed consent was obtained from all participants. Informed parental consent was obtained for participants younger than 20 years. We initially enrolled 349 children under the age of 20 years and 236 adults aged 20–59 years. Following application of the exclusion criteria, 278 children and 199 adults were analyzed. The distributions of refractive errors, mean age, and gender are given in Table 1.

Visitors to the eye clinics were invited to fill out questionnaires, which included the Pittsburgh Sleep Quality Index (PSQI)^44^ and the Hospital Anxiety and Depression Scale (HADS)^45^. Each questionnaire was self-administered and performed from January through March, 2014. The PSQI and HADS scores were calculated according to separate algorithms and then analyzed. The normal range for sleep and mood habits is less than 6 for PSQI^44^ and less than 10 for HADS^45^. Chronotype (morningness/eveningness) was evaluated based on two representative questions from established questionnaires (morningness/eveningness questionnaire)^46^, with possible scores ranging from 10 (far morningness) to 0 (far eveningness).

Eye clinic visitors without visual problems and aged from 10 to 59 years were recruited for testing of visual acuity and eye health, and for pre-examination prior to contact lens or spectacles prescription. Exclusion criteria were dry eye disease (using eyedrops for diagnosed dry eye disease), glaucoma (mean deviation < −12 dB in either eye), cataract (significant lens opacity interfering with optical axis), hyperopia ( ≥ + 3.00^D^), and best corrected visual acuity < 20/30 in both eyes. These ocular comorbidities are known risk factors for sleep and mood disorders^47^ and thus might bias the study results.

Comprehensive ophthalmic examinations were performed by board-certified ophthalmologists and certified orthoptists. Visual acuity was examined after refractive examination with an auto-refractometer. Myopic refractive error was classified as high myopia (≤ −6.00^D^), mild myopia (−5.75^D^ to −0.50^D^), and no myopia (−0.25^D^ to + 2.75^D^) according to a spherical equivalent of the higher myopic eye.

Where appropriate, data are given as the mean ± standard deviation and were analyzed using t-test, ANOVA, Mann-Whitney U test, or Kruskal-Wallis tests. Subscales were analyzed for the PSQI and HADS scores among the 10 to 19-year-old group. Correlations were evaluated using regression analysis. We also compared parameters between contact lens users and non-users. All analyses were performed using StatFlex (Atech, Osaka, Japan) and SPSS version 21 (SPSS Inc., Chicago, IL), with *P* < 0.05 considered significant.

## Results

**Figure 2:**
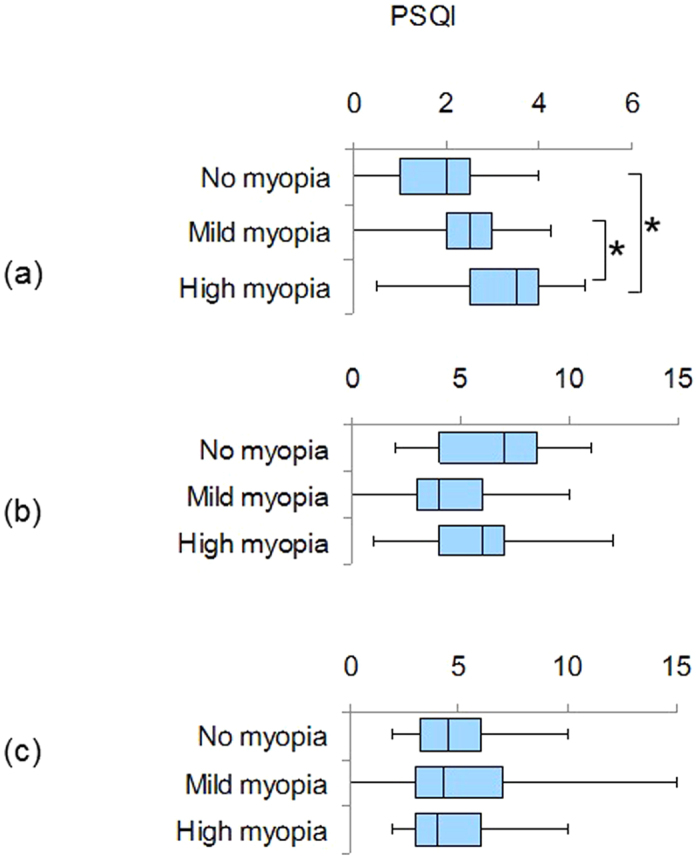
Box plots showing the distribution of Pittsburgh Sleep Quality Index (PSQI) global score in the 10–19 y/o group (a), 20–39 y/o group (b), and 40–59 y/o group (c). Note that highly myopic children are poor sleepers with statistical significance compared with the mild myopia and non-myopia groups (*P < 0.05, Mann-Whitney *U* test after Bonferroni correction). The horizontal line in each diagram indicates the median score for PSQI. The height, positive error bar, and negative error bar of each box indicate the 25th–75th percentiles, maximum values, and minimum values, respectively.

Children in the high myopia group exhibited the poorest PSQI scores (*P* < 0.01, Kruskal-Wallis test, Mann-Whitney U test with Bonfferoni correction), and rated more towards far-eveningness (*P* < 0.05) (Table 2 and Fig. 2). Thus, this group showed the highest probability of sleep (PSQI > 5) and mood (HADS > 9) disorders (Table 2). The same correlations to myopic error were not present among the older patients with myopia except for poor HADS scores in the high myopia group aged 20–39 years (*P* < 0.001). Gender difference was not a significant factor in any age group.

**Figure 3:**
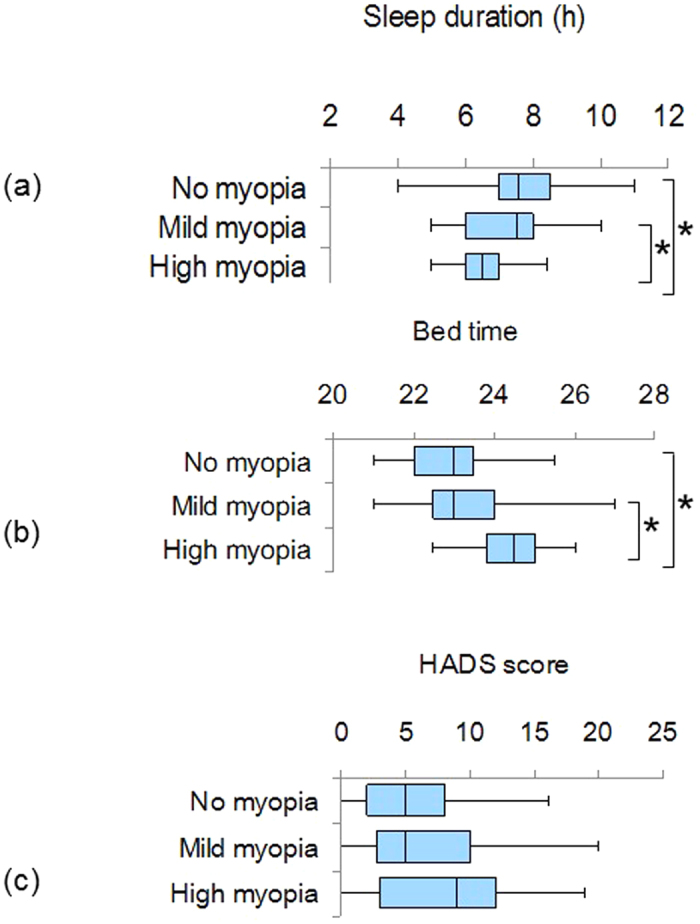
Box plots showing the distribution of sleep duration (a), bedtime (b), and HADS score (c) in children. Note that the highly myopic children were poorer sleepers compared with the less myopic groups (*P < 0.05, Mann-Whitney *U* test after Bonferroni correction). The horizontal line in each diagram indicates the median scores. The height, positive error bar, and negative error bar of each box indicate the 25–75th percentiles, maximum values, and minimum values, respectively.

Subscale analyses of PSQI and HADS in children disclosed the shortest sleep duration in the high myopia group (*P* < 0.01, Mann-Whitney U test with Bonferroni correction; Table 3 and Fig. 3), together with the worst subjective sleep score (*P* < 0.001) and latest bedtime (*P* < 0.05) (Fig. 3).

The regression analyses of correlation between myopic errors and psychiatric indices in children revealed that high myopic errors were significantly correlated with poor PSQI scores (*P* < 0.05), short sleep duration (*P* < 0.01), and late bedtime (*P* < 0.01) (Table 4).

**Figure 4:**
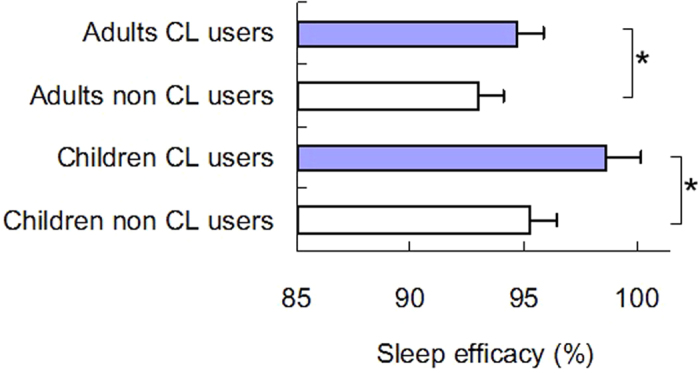
Sleep efficacy of contact lens users. Sleep efficacy was significantly better in contact lens users both for children (age, 15–19 years) and adults (age, 20–39 years). *P < 0.05, Mann-Whitney *U* test.

Sleep and mood indices tested in this study were generally better among contact lens users than among non-users including a non-significant difference for PSQI global score (*P* = 0.06) (Table 5). Sleep efficacy (*P* < 0.05) (Fig. 4) and daytime dysfunction (*P* < 0.05) were significantly better for users than non-users in children aged from 15 to 19 years, while users in all age groups rated more frequently towards morningness on the chronotype scale than the respective non-users (*P* < 0.05, unpaired t test). Sleep efficacy (*P* < 0.05) and sleep difficulty (*P* < 0.05) were also better in adults with contact lens aged from 20 to 39 years than in those of the same age range without contact lens. Children younger than 15 years and adults older than 40 years included very few contact lens users and those age groups were excluded from this analysis.

## Discussion

Our results indicated that myopic children are late and short sleepers. For example, the high myopic children in this study went to bed approximately one hour (74 minutes) later than non-myopes and had one hour less sleep. Such sleep habits could affect systemic and ocular health if continued for several years, because sleep duration is closely related to health and growth in adolescence^48^. In addition, both myopia and sleep disorders are very common in children, thus appropriate health education is recommended at home and in schools, as is regulating the level of environmental light pollution^49^.

Factors such as circadian timing and school timing are extremely relevant to the present analysis given that a sample of children aged 10–19 years were studied and a prominent sleep phase delay is typical for this period of the lifespan^37^,^38^,^39^,^40^. This delay occurs when adolescents face early school start times that effectively shorten night-time sleep duration. Notably, the trends for sleep duration and bedtime observed here were consistent with what occurs in normally developing adolescents and large study is definitely necessary to determine whether the associations observed were due to myopia or normal development, although non-myopic children may serve as control and the correlation between myopic errors and late bedtime was significant when adjusted for age and gender.

Due to the cross sectional nature of this study, myopic progression also could not be determined; however, our results suggest that high myopic children should avoid exposure to light late at night in terms of sleep hygiene and limiting myopic progression. This is supported by animal experiments showing that spontaneous myopia was induced in chicks by keeping them awake in a 50-lux light environment^22^. The authors speculated that elevating retinal dopamine activity via signaling from ipRGCs at elevated light levels could provide additional activation of retinal dopaminergic pathways resulting in axial elongation.

Sleep and mood showed no significant association with refractive error in the adults tested herein. Indeed, even non-myopic adults showed substantially less intra-ocular light transmission and photo entrainment than the equivalent children due to a range of factors, including normal age-related loss in lens transparency, systemic comorbidities, systemic medications, and decreased sunlight exposure due to indoor work^50^,^51^. Consequently, adults have many confounding factors affecting sleep quality.

This study also examined sleep and mood quality in contact lens users for the first time. Despite the similar myopic errors and age, some sleep parameters exhibited significant differences between contact lens users and non-users in both children and adults. Contact lenses are superior to spectacles in terms of visual field and optical aberration. Contact lens users might also be less distressed, which could contribute to the differences in sleep quality since the daily disposable contact lenses offered improvements in QOL measures without negatively impacting vision or eye health and benefits of contact lenses were appearance, satisfaction, activities, and peer perceptions compared with spectacles^52^,^53^,^54^. In addition, sleep and mood disorders in contact lens users might have been underestimated in the present study since dry eye patients were excluded. Many contact lens users have dryness and the prevalence of sleep disorder is high in dry eye patients^47^.

Based on previous studies and the present results, we could hypothesize that long-lasting near sightedness^6^, restricted visual field^55^,^56^, and hyperopia^57^ could induce stress in visual recognition and lead to mood disorder since depression is closely associated with sleep disorders^35^. It is also evident that a large proportion of eye clinic visitors suffer visual problems caused by ocular pathologies and refractive errors^47^, and that such problems could lead to sleep deficits. Therefore, in addition to quality care for organic eye diseases, myopia should be adequately corrected and managed by eye health providers to prevent health problems and economic burdens like presbyopia^58^.

Limitations of this study include the refraction examination and potential systemic comorbidities, thus the non-myopic group may have included hyperopic patients since cycloplegic refraction was not performed in all patients. We also evaluated spherical equivalent only and astigmatism should be analyzed in future similar studies. We did not measure daytime exposure to sunlight because outdoor activity time is a potentially confounding factor in the present study since daylight and outdoor activity suppress myopic progression. Clinic-based cross sectional studies are considered inferior to population-based cross sectional studies for evaluating possible risk factors for disease. To minimize selection bias and possible ocular complications in the present study, board-certified ophthalmologists and certified orthoptists examined all participants. In Japan, an annual eye health check is a legal obligation in school and all children must see an ophthalmologist for a final diagnosis if he/she is suspected of eye disease at the screening examination. Thus, we were able to recruit many healthy children from eye clinics for the study. We could not completely exclude dry eye, glaucoma, and systemic comorbidity since we did not perform a detailed medical interview or specific ocular examinations for patients without suggestive complaints or findings. Finally, we should have included the potential confounding factors into the regression analysis, such as educational levels, times spent in outdoor activities, frequency of near work, the familial presence of myopia, and height. Future studies should additionally examine sleep quality by an objective methodology including electroencephalogram and actigraphy in addition to questionnaire.

## Conclusions

Sleep quality in children was significantly correlated with myopic error, with the high myopia group worst affected. Sleep indices were generally better for contact lens users than non-users. The present results suggested that myopic error could potentially impact on the psychological profile of children.

## Additional Information

**How to cite this article**: Ayaki, M. *et al*. Decreased sleep quality in high myopia children. *Sci. Rep.* **6**, 33902; doi: 10.1038/srep33902 (2016).

## Figures and Tables

**Figure 1:**
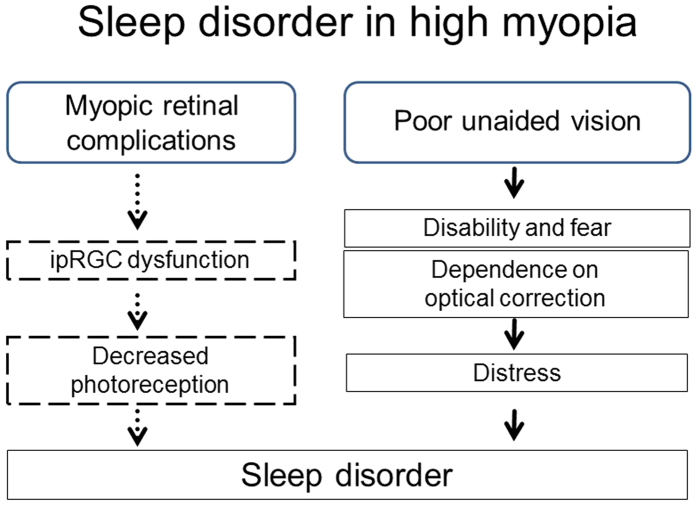
Schematic hypothesis of possible factors contributing to sleep disorders in high myopia. Based on the results of the present study, we hypothesize that sleep disorders in high myopia may be associated with retinal complications and poor unaided vision. High myopia children may suffer blurring and dependence on optical correction including with spectacles and contact lenses. In addition, myopic retina is accompanied by multiple dysfunctions presumably involving intrinsically photosensitive retinal ganglion cells, although evidence is to be further confirmed in human. High myopia children might therefore, consciously or unconsciously, be affected by this distress and disability during both the daytime and night, leading to sleep disorders. ipRGC, intrinsically photosensitive retinal ganglion cell.

**Table 1:** Patient demographics and myopic refractive errors.

Age groups	10–19 years	20–39 years	40–59 years
Total number and gender (% of male)	278 (44.0%)	113 (36.9%)	95 (44.1%)
Mean age (median)	14.2 ± 2.6 (15)	28.9 ± 5.7 (30)	48.3 ± 5.6 (50)
High myopia			
Number and gender (% of male)	21 (42.9%)	23 (21.7%)	19 (52.6%)
Mean age	16.7 ± 2.4	27.6 ± 5.2	48.0 ± 6.1
Spherical equivalent^A^ (diopter)	−7.50 ± 2.68	−8.32 ± 2.81	−8.50 ± 2.19
Mild myopia			
Number and gender (% of male)	156 (41.0%)	75(41.3%)	50 (44.0%)
Mean age	14.3 ± 2.6	28.9 ± 5.6	47.9 ± 6.0
Spherical equivalent^A^ (diopter)	−2.56 ± 1.38	−3.77 ± 1.16	−3.48 ± 1.23
No myopia			
Number and gender (% of male)	101 (48.5%)	15 (26.7%)	26 (38.5%)
Mean age	13.5 ± 2.3	30.7 ± 6.2	49.4 ± 6.0
Spherical equivalent^A^ (diopter)	0.02 ± 0.15	—	—

^A^Spherical equivalent in the higher myopic eye.

**Table 2:** Indices of participants of various myopic levels and age.

Age group and myopic errors	High myopia	Mild myopia	No myopia	Total	*P* value^A^
Age, 10–19 (years)					
PSQI global score	4.52 ± 2.12	3.56 ± 2.58	3.37 ± 2.39	3.51 ± 2.44	*P* < 0.01
Male	4.44 ± 1.59	3.15 ± 2.70	3.24 ± 2.31	3.31 ± 2.37	n.s.
Female	4.58 ± 2.81	3.79 ± 2.50	3.49 ± 2.44	3.67 ± 2.49	n.s.
% of subjects PSQI > 5	22.7	18.9	17.0	18.0	
HADS score	8.29 ± 5.45	7.32 ± 5.46	6.37 ± 5.15	6.78 ± 5.28	n.s.
Male	6.67 ± 6.40	6.31 ± 4.78	6.39 ± 5.13	6.39 ± 5.14	n.s.
Female	9.50 ± 4.52	7.88 ± 5.77	6.34 ± 5.16	7.09 ± 5.38	n.s.
% of subjects HADS > 9	40.9	25.7	22.0	24.5	
Morningness/eveningness	2.50 ± 1.36	3.16 ± 1.91	3.65 ± 1.95	3.43 ± 1.92	P < 0.05
Male	2.78 ± 1.92	3.04 ± 2.01	3.84 ± 2.04	3.58 ± 2.02	n.s.
Female	2.22 ± 1.39	3.23 ± 1.87	3.47 ± 1.85	3.31 ± 1.84	n.s.
Age, 20–39 (years)					
PSQI global score	5.52 ± 3.02	4.68 ± 1.88	6.33 ± 2.71	5.07 ± 2.32	n.s.
Male	5.60 ± 2.07	4.81 ± 2.00	7.50 ± 3.42	5.18 ± 2.26	n.s.
Female	5.50 ± 3.29	4.59 ± 1.80	5.90 ± 2.47	5.01 ± 2.37	n.s.
% of subjects PSQI > 5	47.8	32.0	53.3	38.1	
HADS	10.48 ± 6.52	9.12 ± 5.57	8.00 ± 5.79	9.25 ± 5.80	*P* < 0.001
Male	9.00 ± 6.29	9.11 ± 4.54	5.45 ± 4.27	8.76 ± 4.72	n.s.
Female	10.89 ± 6.70	9.14 ± 6.27	8.81 ± 6.23	9.54 ± 6.23	n.s.
% of subjects HADS > 9	43.5	45.3	6.7	39.8	
Morningness/eveningness	4.26 ± 2.20	3.43 ± 1.90	3.13 ± 1.62	3.56 ± 1.95	n.s.
Male	4.40 ± 2.79	3.48 ± 2.20	3.00 ± 1.41	3.55 ± 2.12	n.s.
Female	4.22 ± 2.10	3.39 ± 1.67	3.18 ± 1.78	3.56 ± 1.72	n.s.
Age, 40–59 (years)					
PSQI global score	4.58 ± 2.29	5.47 ± 3.47	4.80 ± 2.00	5.20 ± 2.86	n.s.
Male	5.60 ± 2.55	5.75 ± 3.91	4.83 ± 1.70	5.45 ± 3.08	n.s.
Female	3.44 ± 1.33	5.27 ± 3.16	5.13 ± 2.25	4.99 ± 2.69	n.s.
% of subjects PSQI > 5	26.3	42.0	34.6	36.8	
HADS	13.11 ± 6.92	11.60 ± 6.79	9.45 ± 5.83	11.22 ± 6.61	n.s.
Male	16.20 ± 5.79	12.25 ± 6.41	8.33 ± 5.63	12.07 ± 6.57	n.s.
Female	9.25 ± 6.54	11.11 ± 7.14	10.19 ± 6.05	10.53 ± 6.63	n.s.
% of subjects HADS > 9	61.1	60.0	46.2	56.4	
Morningness/eveningness	4.11 ± 1.81	4.48 ± 0.81	4.46 ± 0.90	4.42 ± 2.06	n.s.
Male	3.90 ± 2.21	4.45 ± 1.99	5.00 ± 1.95	4.46 ± 1.98	n.s.
Female	4.38 ± 1.99	4.50 ± 2.39	4.19 ± 2.01	4.38 ± 2.14	n.s.

Unless indicated otherwise, data are given as the mean ± SD. ^A^Kruskal-Wallis test.

PSQI, Pittsburgh sleep quality index; HADS, Hospital anxiety and depression scale; n.s., not significant.

**Table 3:** Subscales of PSQI and HADS in children.

Myopic grade	High myopia	Mild myopia	No myopia	All patients	*P* value^A^
Sleep latency	0.90 ± 1.07	0.77 ± 0.94	0.82 ± 0.86	0.81 ± 0.89	n.s.
Male	1.00 ± 1.12	0.67 ± 0.92	0.80 ± 0.79	0.79 ± 0.84	
Female	0.82 ± 1.08	0.83 ± 0.95	0.83 ± 0.91	0.83 ± 0.93	
Sleep efficacy	0.05 ± 0.22	0.12 ± 0.43	0.11 ± 0.41	0.11 ± 0.40	n.s.
Male	0.11 ± 0.33	0.07 ± 0.27	0.11 ± 0.46	0.10 ± 0.41	
Female	0.00 ± 0.00	0.15 ± 0.51	0.12 ± 0.35	0.12 ± 0.39	
Sleep duration	1.29 ± 0.78	0.69 ± 0.92	0.53 ± 0.84	0.68 ± 0.84	*P* < 0.01
Male	1.00 ± 0.87	0.44 ± 0.80	0.48 ± 0.75	0.51 ± 0.77	
Female	1.50 ± 0.69	0.96 ± 0.94	0.69 ± 0.81	0.81 ± 0.87	
Subjective sleep	1.25 ± 0.55	0.77 ± 0.69	0.85 ± 0.66	0.86 ± 0.67	*P* < 0.001
Male	1.11 ± 0.33	0.70 ± 0.78	0.84 ± 0.70	0.83 ± 0.70	
Female	1.36 ± 0.67	0.81 ± 0.64	0.87 ± 0.63	0.88 ± 0.64	
Sleep difficulty	0.50 ± 0.51	0.44 ± 0.50	0.40 ± 0.51	0.42 ± 0.51	n.s.
Male	0.67 ± 0.50	0.52 ± 0.51	0.45 ± 0.52	0.48 ± 0.52	
Female	0.36 ± 0.51	0.40 ± 0.49	0.35 ± 0.50	0.37 ± 0.50	
Daytime dysfunction	0.81 ± 0.95	0.65 ± 0.81	0.51 ± 0.76	0.61 ± 0.79	n.s.
Male	0.56 ± 0.53	0.74 ± 0.98	0.54 ± 0.73	0.58 ± 0.78	
Female	1.00 ± 1.12	0.60 ± 0.71	0.60 ± 0.79	0.63 ± 0.80	
HADS-A	4.81 ± 3.46	4.64 ± 3.42	3.70 ± 3.12	4.04 ± 3.25	n.s.
Male	3.89 ± 4.31	3.89 ± 2.86	3.58 ± 3.03	3.67 ± 3.08	
Female	5.50 ± 2.65	5.01 ± 3.66	3.81 ± 3.22	4.34 ± 3.37	
HADS-D	3.48 ± 2.56	2.89 ± 2.61	2.66 ± 2.75	2.79 ± 0.66	n.s.
Male	2.78 ± 2.54	2.42 ± 2.76	2.80 ± 3.00	2.71 ± 2.90	
Female	4.00 ± 2.56	3.15 ± 2.52	2.53 ± 2.50	2.84 ± 2.53	

^A^*P* < 0.05, Kruskal-Wallis test for three age groups. Notes: Data are mean ± standard deviation. Abbreviations: PSQI, Pittsburgh Sleep Quality Index; HADS, Hospital Anxiety and Depression Scale; HADS-A, anxiety subscale in HADS; HADS-D, depression subscale in HADS; n.s., not significant.

**Table 4:** Stepwise regression analysis between myopic errors^A^ and psychiatric parameters in children.

	PSQI global score		Sleep duration		Bed time		HADS score	
	β	*P*-value	β	*P*-value	β	*P*-value	β	*P*-value
Simple regression	0.147	0.016*	−0.194	0.001*	0.279	0.000*	0.071	0.244
Multiple regression^B^	0.050	0.433	−0.064	0.385	0.186	0.022*	0.017	0.786

HADS = Hospital Anxiety and Depression Scale; PSQI = Pittsburgh Sleep Quality Index.

*P < 0.05.

^A^High myopia = 2, mild myopia = 1, no myopia = 0.

^B^Adjusted for age and gender.

**Table 5:** Indices of contact lens users and non users for myopic correction.

Age group and parameters	Contact lens users	Non users	*P* value^A^
Age, 15–19 (years)			
#	38	94	
Age, % of male	16.0 ± 2.1, 31.6%	16.3 ± 1.7, 42.1%	n.s
Myopic error (diopter)	−2.75 ± 2.00	−3.13 ± 2.85	n.s.
PSQI global score	3.87 ± 2.59	4.59 ± 0.28	n.s. (0.06)
Sleep latency	0.76 ± 0.91	0.92 ± 0.63	n.s.
Sleep duration	1.03 ± 0.21	1.12 ± 0.63	n.s.
Sleep efficacy	0.05 ± 0.28	0.19 ± 0.42	*P* < 0.05
Sleep difficulty	0.34 ± 0.56	0.45 ± 0.35	n.s.
Daytime dysfunction	0.63 ± 0.14	0.90 ± 0.70	*P* < 0.05
Subjective sleep	1.00 ± 0.12	0.97 ± 0.49	n.s.
HADS score	7.13 ± 0.85	8.14 ± 3.79	n.s.
HADS-A	4.40 ± 0.52	4.80 ± 0.44	n.s.
HADS-D	2.74 ± 2.59	3.43 ± 1.96	n.s.
Morningness/eveningness	3.34 ± 0.29	2.85 ± 1.33	*P* < 0.05
Age, 20–39 (years)			
#	52	60	
Age, % of male	27.5 ± 5.1, 42.3	30.0 ± 5.9, 30.0	*P* < 0.05
Myopic error (diopter)	−5.18 ± 2.66	−6.10 ± 3.66	n.s.
PSQI global score	4.81 ± 2.03	5.30 ± 1.61	n.s.
Sleep latency	0.77 ± 0.84	0.97 ± 0.63	n.s.
Sleep duration	1.42 ± 0.84	1.25 ± 0.63	n.s.
Sleep efficacy	0.15 ± 0.49	0.36 ± 0.49	*P* < 0.05
Sleep difficulty	0.48 ± 0.49	0.79 ± 0.43	*P* < 0.001
Daytime dysfunction	0.73 ± 0.77	0.61 ± 0.42	n.s.
Subjective sleep	1.15 ± 0.63	1.31 ± 0.42	n.s.
HADS score	9.30 ± 4.90	9.21 ± 4.20	n.s.
HADS-A	5.50 ± 3.96	5.31 ± 2.22	n.s.
HADS-D	3.81 ± 2.59	3.90 ± 2.38	n.s.
Morningness/eveningness	3.71 ± 1.89	3.43 ± 1.33	n.s.

Unless indicated otherwise, data are given as the mean ± SD. ^B^Kruskal-Wallis test.

PSQI, Pittsburgh sleep quality index; HADS, Hospital anxiety and depression scale; HADS-A, anxiety subscale in Hospital Anxiety and Depression Scale; HADS-D, depression subscale in HADS; n.s., not significant.
